# Simultaneous Parameter Learning and Bi-clustering for Multi-Response Models

**DOI:** 10.3389/fdata.2019.00027

**Published:** 2019-08-14

**Authors:** Ming Yu, Karthikeyan Natesan Ramamurthy, Addie Thompson, Aurélie C. Lozano

**Affiliations:** ^1^Booth School of Business, The University of Chicago, Chicago, IL, United States; ^2^IBM Research, Yorktown Heights, NY, United States; ^3^Department of Plant, Soil and Microbial Sciences, Michigan State University, East Lansing, MI, United States; ^4^Department of Agronomy, Purdue University, West Lafayette, IN, United States

**Keywords:** high-throughput phenotyping, multitask learning, convex clustering, bi-clustering, sparse linear regression, genome-wide association studies

## Abstract

We consider multi-response and multi-task regression models, where the parameter matrix to be estimated is expected to have an unknown grouping structure. The groupings can be along tasks, or features, or both, the last one indicating a bi-cluster or “checkerboard” structure. Discovering this grouping structure along with parameter inference makes sense in several applications, such as multi-response Genome-Wide Association Studies (GWAS). By inferring this additional structure we can obtain valuable information on the underlying data mechanisms (e.g., relationships among genotypes and phenotypes in GWAS). In this paper, we propose two formulations to simultaneously learn the parameter matrix and its group structures, based on convex regularization penalties. We present optimization approaches to solve the resulting problems and provide numerical convergence guarantees. Extensive experiments demonstrate much better clustering quality compared to other methods, and our approaches are also validated on real datasets concerning phenotypes and genotypes of plant varieties.

## 1. Introduction

**Figure 1:**
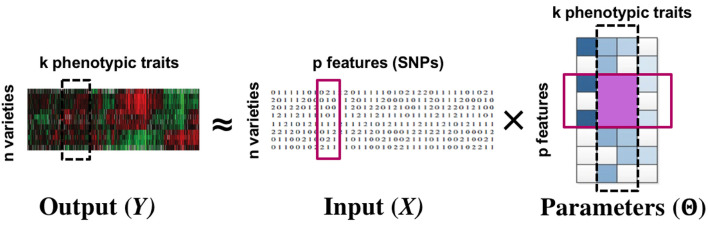
Multi-response GWAS: the simultaneous grouping relationship between phenotypic traits and SNPs manifest as a block structure (row + column groups) in the parameter matrix. The row and column groups are special cases of the more general block structure. Our proposed approach infers the parameter matrix as well as the group structures.

We consider multi-response and multi-task regression models, which generalize single-response regression to learn predictive relationships between multiple input and multiple output variables, also referred to as tasks (Borchani et al., 2015). The parameters to be estimated form a matrix instead of a vector. In several applications, there exist joint group relationships between inputs and outputs. A motivating example is that of multi-response Genome-Wide Association Studies (GWAS) (Schifano et al., 2013), where for instance a group of Single Nucleotide Polymorphisms or SNPs (input variables or features) might influence a group of phenotypes (output variables or tasks) in a similar way, while having little or no effect on another group of phenotypes. Similarly, as another example, stocks values of related companies can affect the future value of a group of stocks similarly. In such cases, the model parameters belonging to the same input-output group tend to be close to each other, and it is desirable to *uncover* and *exploit* these structures in estimating the parameter matrix. See Figure 1 for an example.

### 1.1. Contributions

In this work, we develop formulations that *simultaneously* learn: (a) the parameters of multi-response/task regression models, and (b) the grouping structure in the parameter matrix (row or column or both) that reflects the group relationship between inputs and outputs. We present optimization approaches to efficiently solve the resulting convex problems, and show their numerical convergence. We describe and justify several hyperparameter choices we make during this optimization. Our methods are validated empirically on synthetic data and on real-world datasets concerning phenotypes and genotypes of plant varieties. From the synthetic data experiments, we find that our methods provide a much better and more stable (i.e., lesser standard error) recovery of the underlying group structure. In real-world data experiments, our approaches reveal natural groupings of phenotypes and *checkerboard* patterns of phenotype-SNP groups that inform us of the joint relationship between them.

We emphasize that the parameters as well as the grouping structures are *fully unknown a-priori*, and inferring them simultaneously is our major contribution. This is in contrast to the naive way of estimating the parameters first and then clustering. This naive approach has the danger of propagating the estimation error into clustering, particularly in high dimensions, where the estimator is usually inaccurate due to lack of sufficient samples. Moreover, the clustering step of the naive approach does not use the full information of the data. The joint estimation-clustering procedure we propose naturally promotes sharing of information within groups. Our formulations adopt the convex bi-clustering cost function (Chi et al., 2014) as the regularizer to encourage groupings between columns (tasks) and rows (features) in the parameter matrix. Note that, Chi et al. (2014) assume that the data matrix to be used for bi-clustering is known a-priori, which is obviously not the case for our setting. As a result, Chi et al. (2014) can deal only with fixed data and cannot estimate unknown model parameters, while our approaches can simultaneously estimate parameters and discover the clustering structure in them.

To the best of our knowledge, this is the first method that can simultaneously cluster and estimate the parameters efficiently in a unified optimization. *We emphasize that our main goal is to discover the underlying parameter bi-cluster structure without compromising estimation accuracy*. Experiments show that our clusterings are better than other methods while the estimation accuracy is no worse or sometimes even better.

### 1.2. Related Work

The premise in multi-task learning is that appropriate sharing of information can benefit all the tasks (Caruana, 1998; Obozinski et al., 2006; Yu et al., 2018). Assuming all tasks to be closely related can be excessive as it ignores the underlying specificity of the mappings. There have been several extensions to multi-task learning that address this problem. The authors in Jalali et al. (2010) propose a *dirty* model for feature sharing among tasks, wherein a linear superposition of two sets of parameters—one that is common to all tasks, and one that is task-specific—are used. Kim and Xing (2010) leverages a *predefined* tree structure among the output tasks (e.g., using hierarchical agglomerative clustering) and imposes group regularizations on the task parameters based on this tree. The approach proposed in Kumar and Daume (2012) learns to share by defining a set of *basis task parameters* and posing the task-specific parameters as a sparse linear combination of these. Jacob et al. (2009) and Kang et al. (2011) assume that the tasks are clustered into groups and proceed to learn the group structure along with the task parameters using a convex and an integer quadratic program, respectively. However, these approaches do not consider joint clustering of the features. In addition, the mixed integer program of Kang et al. (2011) is computationally intensive and greatly limits the maximum number of tasks that can be considered. Another pertinent approach is the Network Lasso formulation presented in Hallac et al. (2015). This formulation, however, is limited to settings where only clustering among the tasks is needed.

As mentioned before, convex bi-clustering method (Chi et al., 2014) aims at grouping observations and features in a data matrix; while our approaches aim at discovering groupings in the parameter matrix of multi-response regression models while jointly estimating such a matrix, and the discovered groupings reflect groupings in features and responses.

### 1.3. Roadmap

In section 2, we will discuss the proposed joint estimation-clustering formulations; in section 3, we will present the optimization approaches. The choice of hyperparameters used and their significance is discussed in section 4. We illustrate the solution path for one of the formulations in section 4.3. We will provide results for estimation with synthetic data, and two case studies using multi-response GWAS with real data in sections 5, 6, respectively. We conclude in section 7. Additional details and convergence proofs for the optimization approaches are provided in the Supplementary Material.

## 2. Problem Statement and Proposed Methods

We will motivate and propose two distinct formulations for simultaneous parameter learning and clustering with general supervised models involving matrix valued parameters. Our formulations will be developed around multi-task regression in this paper. We are interested in accurate parameter estimation as well as understanding the *bi-cluster* or *checkerboard* structure of the parameter matrix. More formally, denote by *Z* the observed data, Θ the model parameters to be estimated, and *L*(*Z*; Θ) a general loss function, and *R*(Θ) to be the regularization.

In multi-task regression, *Z* = {*X, Y*} where $X_{s}\in {\mathbb{R}}^{n\times p}$ are the design matrices and $Y_{s}\in {\mathbb{R}}^{n}$ are the response vectors for each task *s* = 1, …, *k*. Θ is a matrix in ℝ^*p* × *k*^ containing the regression coefficients for each task. A popular choice for *L* is the squared loss: $L\left(Z;\Theta \right)=\overset{k}{\underset{s=1}{\sum }}\|Y_{s}-X_{s}\Theta_{s}{\|}_{2}^{2}$. For regularization *R*(Θ), the ℓ_1_ norm, denoted as ||Θ||_1_, is commonly used. Here we wish to discover the bi-cluster structure among features and responses, respectively the rows and columns of Θ.

### 2.1. Formulation 1: “Hard Fusion”

We begin with the simplest formulation, which, as we shall see, is a special case of the latter one.

(1) $$\begin{array}{r} \underset{\Theta }{\min}L\left(Z;\Theta \right)+\lambda_{1}R\left(\Theta \right)+\lambda_{2}[\Omega_{W}\left(\Theta \right)+\Omega_{\tilde{W}}\left(\Theta^{T}\right)]. \end{array}$$

Here *L*(*Z*; Θ) is the loss function, *R*(Θ) is a regularizer, and $\Omega_{W}\left(\Theta \right)=\underset{i<j}{\sum }w_{ij}\|\Theta_{\cdot i}-\Theta_{\cdot j}{\|}_{2}$ and Θ_·*i*_ is the *i*th column of Θ. Ω_*W*_(Θ) is inspired by the convex bi-clustering objective (Chi et al., 2014, Equation 1) and it encourages sparsity in differences between columns of Θ. Similarly, $\Omega_{W}\left(\Theta^{T}\right)$ encourages sparsity in the differences between the rows of Θ. When the overall objective is optimized, we can expect to see a checkerboard pattern in the model parameter matrix. Note that *W* and $\tilde{W}$ are non-negative weights that reflect our prior belief on the closeness of the rows and columns of Θ.

**Figure 2:**
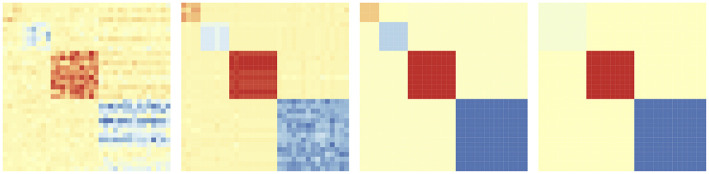
Evolution of the bi-clustering structure of model coefficient matrix Θ as regularization parameter λ_2_ increases.

The degree of *sharing* of parameters and hence that of bi-clustering, is controlled using the tuning parameter λ_2_. When λ_2_ is small, each element of Θ will be its own bi-cluster. As λ_2_ increases, more elements of Θ *fuse* together, the number of rectangles in the checkerboard pattern will reduce. See Figure 2 for the change of the checkerboard structure as λ_2_ increases. Further, by varying λ_2_ we get a solution path instead of just a point estimate of Θ (see section 4.3). In the rest of the paper, we will use the same design matrix *X* across all tasks for simplicity, without loss of generality.

For sparse multi-task linear regression, we have *L*(*Z*; Θ) = *L*(*X, Y*; Θ) and formulation 1 can be instantiated as,

(2) $$\begin{array}{r} \underset{\Theta }{\min}\|Y-X\Theta {\|}_{F}^{2}+\lambda_{1}\overset{k}{\underset{i=1}{\sum }}\|\Theta_{\cdot i}{\|}_{1}+\lambda_{2}[\Omega_{W}\left(\Theta \right)+\Omega_{\tilde{W}}\left(\Theta^{T}\right)]. \end{array}$$

Here the rows of Θ correspond to the features, i.e., the columns of *X*, and the columns of Θ correspond to the tasks, i.e., the columns of *Y*. Therefore, the checkerboard pattern in Θ provides us insights on the groups of features that go together with the groups of tasks.

### 2.2. Formulation 2: “Soft Fusion”

Formulation 1 is natural and simple, but it forces the parameters belonging to the same row or column cluster to be equal, and this may be limiting. To relax this requirement, we introduce a surrogate parameter matrix Γ that will be used for bi-clustering. This will be mandated to be close to Θ. For multitask regression this yields the objective

(3) $$\begin{array}{r} \underset{\Theta ,\Gamma }{\min}\|Y-X\Theta {\|}_{F}^{2}+\lambda_{1}\overset{k}{\underset{i=1}{\sum }}\|\Theta_{\cdot i}{\|}_{1}+\lambda_{2}\overset{k}{\underset{i=1}{\sum }}\|\Theta_{\cdot i}-\Gamma_{\cdot i}{\|}_{2}^{2}\\ +\lambda_{3}\left[\Omega_{W}\left(\Gamma \right)+\Omega_{\tilde{W}}\left(\Gamma^{T}\right)\right]. \end{array}$$

**Remark 1**. To interpret this more carefully, let us assume that $\Theta_{\cdot i}=\bar{\Theta_{\cdot i}}+\Gamma_{\cdot i}$ in Equation (3). In other words, Θ_·*i*_ has a global component $\bar{\Theta_{\cdot i}}$, and the component Γ_·*i*_ that participates in the clustering. As λ_2_ → ∞, $\bar{\Theta_{\cdot i}}\to 0$, and hence Θ_·*i*_ → Γ_·*i*_. Now, formulation 2 reduces to formulation 1. Further, if λ_1_ and λ_2_ are held constant while only λ_3_ increases, $\Theta_{\cdot i}\to \bar{\Theta_{\cdot i}}+\Gamma$, since Γ_·*i*_ → Γ for all *i*. The key difference between formulation 2 and 1 is the presence of a task-specific global component $\bar{\Theta_{\cdot i}}$, which lends additional flexibility in modeling the individual tasks even when λ_3_ → 0. Whereas, in Equation (2), when λ_2_ → ∞, all the components of Θ_·*i*_ take the same value for all *i*, and the tasks are forced to share the same coefficients without any flexibility.

**Remark 2**. In certain applications, it might make sense to cluster together features/tasks whose effects have the same amplitude but different signs. This can be accommodated by considering $\Omega_{W}\left(\Theta \right)=\underset{i<j}{\sum }w_{ij}\|\Theta_{\cdot i}-c_{ij}\Theta_{\cdot j}{\|}_{2}$ where *c*_*i,j*_ ∈ {−1, 1} are predefined constants reflecting whether the features or tasks are expected to be negatively or positively correlated.

## 3. Optimization Algorithms for the Proposed Formulations

We describe the optimization procedures to solve the two proposed formulations. Note that as long as the loss function *L*(*X, Y*; Θ) and the regularization *R*(Θ) are convex, our formulations are also convex in Θ and Γ, and hence can be solved using modern convex optimization approaches. Here we adopt two computationally efficient approaches.

### 3.1. Optimization for Formulation 1

For our formulation 1 we use the proximal decomposition method introduced in Combettes and Pesquet (2008). This is an efficient algorithm for minimizing the sum of several convex functions. Our general objective function (1) involves three such functions: *f*_1_ being *L*(*X, Y*; Θ), *f*_2_ being *R*(Θ), and *f*_3_ being the term that multiplies λ_2_. At a high level, the algorithm iteratively applies proximal updates with respect to these functions until convergence.

We stack the regression matrix Θ into a column vector $\left(\Theta_{1};\ldots ;\Theta_{k}\right)\in {\mathbb{R}}^{pk}$. The proximal operator is given by: ${\text{prox}}_{f}b=\underset{a}{\text{argmin}}(f\left(a\right)+\frac{1}{2}\|b-a{\|}_{2}^{2})$, where *a* and *b* are *pk*-dimensional vectors. The proximal operator of the regularized loss can be computed according to the specific *L* and *R* functions. The overall optimization procedure is given in **Algorithm 1** and with the following update rules.

- Update for $f_{1}=\|Y-X\Theta {\|}_{F}^{2}$: Let (*a*_1_; …; *a*_*k*_) = prox_σ*f*_1__(*b*_1_; …; *b*_*k*_), For each *s* ∈ {1, …, *k*}, we have
  $$a_{s}={\left(\sigma X_{s}^{T}X_{s}+\frac{1}{2}I_{p}\right)}^{-1}\cdot \left(\sigma X_{s}^{T}y_{s}+\frac{1}{2}b_{s}\right).$$
  This step corresponds to the closed-form formula of a ridge regression problem. For very large *p* we can employ efficient approaches, such as Lu and Foster (2014) and McWilliams et al. (2014).
- Update for $f_{2}=\lambda_{1}\overset{k}{\underset{i=1}{\sum }}\|\Theta_{\cdot i}{\|}_{1}$: Let (*a*_1_; …; *a*_*k*_) = prox_σ*f*_2__(*b*_1_; …; *b*_*k*_), For each *s* ∈ {1, …, *k*}, *j* ∈ {1, …, *p*},
  $${\left[a_{s}\right]}_{j}={\left[1-\frac{\lambda_{1}\sigma }{\left|{\left[b_{s}\right]}_{j}\right|}\right]}_{+}\cdot {\left[b_{s}\right]}_{j}.$$
- Updates for $f_{3}=\lambda_{2}[\Omega_{W}\left(\Theta \right)+\Omega_{\tilde{W}}\left(\Theta^{T}\right)]$: This is the standard bi-clustering problem on Θ and can be solved efficiently using the COnvex BiclusteRing Algorithm (COBRA) introduced in Chi et al. (2014), and described in Algorithm 3 (Supplementary Material) for completeness.

**Algorithm 1:** Proximal decomposition for formulation 1.

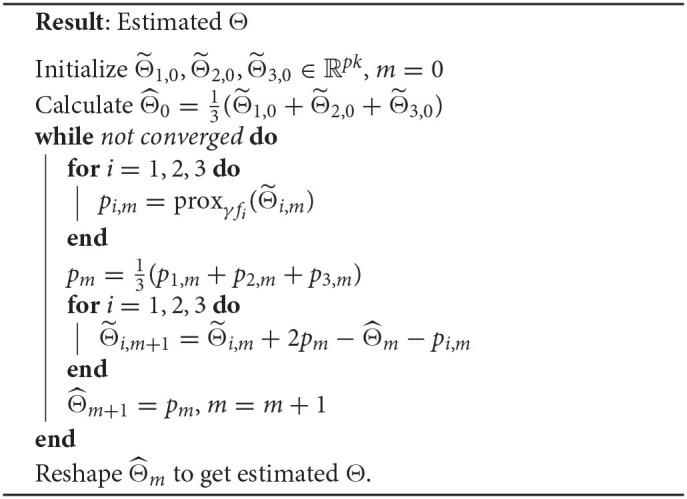

### 3.2. Optimization for Formulation 2

For our formulation 2 we use an alternating minimization method on Θ and Γ; i.e., we alternatively minimize over Θ and Γ with the other fixed. The first alternating step is to estimate Θ while fixing Γ. This minimization problem is separable for each column and each sub-problem can be easily written as a standard Lasso problem:

(4) $$\begin{array}{r} \underset{\Theta_{\cdot i}}{\min}\|{\tilde{y}}_{i}-\tilde{X}\Theta_{\cdot i}{\|}_{2}^{2}+\lambda_{1}\|\Theta_{\cdot i}{\|}_{1} \end{array}$$

by defining ${\tilde{y}}_{i}=]y_{i},\sqrt{\lambda_{2}}\Gamma_{\cdot i}]$ and $\tilde{X}=\left[X,\sqrt{\lambda_{2}}I_{p}\right]$ and hence can be solved efficiently and in parallel for each column. In the second step, we fix Θ and optimize for Γ. The optimization is

(5) $$\begin{array}{r} {\text{minimize}}_{\Gamma }\overset{k}{\underset{i=1}{\sum }}\|\Theta_{\cdot i}-\Gamma_{\cdot i}{\|}_{2}^{2}+\frac{\lambda_{3}}{\lambda_{2}}\left[\Omega_{W}\left(\Gamma \right)+\Omega_{\tilde{W}}\left(\Gamma^{T}\right)\right] \end{array}$$

which is a standard bi-clustering problem on Θ and can be solved efficiently using the COnvex BiclusteRing Algorithm (COBRA) introduced in Chi et al. (2014), and described in Algorithm 3 (Supplementary Material) for completeness. The overall procedure is given in **Algorithm 2**.

**Algorithm 2:** Alternating minimization for formulation 2.

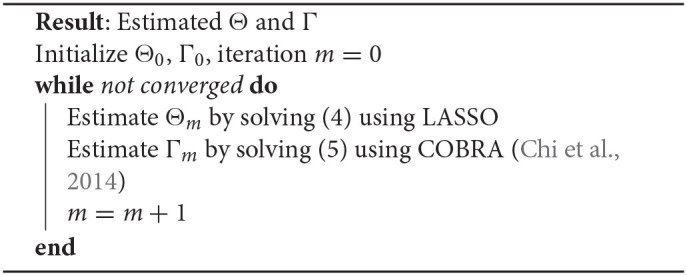

### 3.3. Numerical Convergence

We establish the following convergence result for our algorithms, when the loss function *L*(*X, Y*; Θ) is convex in Θ. The proofs are given in the Supplementary Material.

Proposition 1. *The algorithm described in section 3.1 converges to the global minimizer.*

Proposition 2. *The algorithm described in section 3.2 converges to the global minimizer.*

For both formulations 1 and 2, computational complexity is dominated by the use of the COBRA algorithm. COBRA solves a sequence of convex clustering problems. The subroutine used to solve each convex clustering subproblem scales in storage and computational operations as O(*kpq*), where *k* is the number of tasks, *p* is the number of features and *q* is the number of non-zero weights. In our case *q* is much smaller than *p*^2^ and *k*^2^. Indeed as we shall see in section 4.1, our weights are based on κ = 5 nearest neighbors.

## 4. Hyperparameter Choices, Solution Path, and Variations

We describe and justify the various hyperparameters choices for formulations 1 and 2.

### 4.1. Weights and Sparsity Regularization

The choice of the column and row similarity weights *W* and $\tilde{W}$ can affect the quality of the clustering results and we follow the suggestion in Chi et al. (2014) to set these. However, in their case the data matrix to be clustered is fixed and known, but in our case, the coefficient matrix Θ we want to cluster is not known. We will get a rough estimate $\hat{\Theta }$ by just minimizing the regularized loss function *L*(*Z*; Θ) + λ_1_*R*(Θ). For example, with multi-task regression in Equations (2) and (3), we can solve

(6) $$\begin{array}{r} \underset{\Theta }{\min}\|Y-X\Theta {\|}_{F}^{2}+\lambda_{1}\overset{k}{\underset{i=1}{\sum }}\|\Theta_{\cdot i}{\|}_{1}, \end{array}$$

where λ_1_ is tuned by cross-validation (CV) and re-used in the rest of the algorithm. From our multi-task regression experiment, we find that the clustering results are robust to the choice of λ_1_.

With the estimated $\hat{\Theta }$ we are ready to compute *W* and $\tilde{W}$. The weights for the columns *i* and *j* are computed as $w_{ij}=1_{ij}^{k}\cdot \exp(-\phi \|{\hat{\Theta }}_{\cdot i}-{\hat{\Theta }}_{\cdot j}{\|}_{2}^{2})$ where $1_{ij}^{k}$ is 1 if *j* is among *i*'s κ-nearest-neighbors or vice versa and 0 otherwise. Here ϕ is non-negative and ϕ = 0 corresponds to uniform weights. In our synthetic and real data experiments we fix ϕ = 20. $\tilde{W}$ is computed analogously. It is important to keep the two penalty terms Ω_*W*_(Θ) and $\Omega_{\tilde{W}}\left(\Theta^{T}\right)$ on the same scale, else the row or column objective will dominate the solution. We normalize so that the column weights sum to $1/\sqrt{n}$ and the row weights sum to $1/\sqrt{p}$.

### 4.2. Penalty Multiplier Tuning

We set the penalty multipliers (λ_1_, λ_2_, and λ_3_) for both the formulations using a CV approach. We randomly split our samples into a training set and a hold-out validation set, fitting the models on the training set and then evaluating the root-mean-squared error (RMSE) on the validation set to choose the best values. In order to reduce the computational complexity, we estimate the multipliers greedily, one or two at a time. From our simulations, we determined that this is a reasonable choice. We recognize that these can be tuned further on a case-by-case basis.

λ_1_ is set to the reasonable value as determined in section 4.1 for both formulations, since the clustering results are robust to this choice. For formulation 1, we estimate the best λ_2_ by CV using Equation (1). For formulation 2, the tuning process is similar, but we pick a sequence of λ_2_ and λ_3_. We estimate both ${\hat{\Theta }}_{\lambda_{2},\lambda_{3}}$ and ${\hat{\Gamma }}_{\lambda_{2},\lambda_{3}}$, but calculate RMSE with ${\hat{\Gamma }}_{\lambda_{2},\lambda_{3}}$, since it is used in the clustering objective. When the path of bi-clusterings is computed, we fix λ_2_ to the CV estimate and vary only λ_3_.

### 4.3. Solution Paths

One can obtain the entire solution paths for the estimated coefficients Θ by varying the penalty multipliers. Here we provide an example using a synthetic dataset generated as follows. We consider the multi-task regression model: *Y* = *X*Θ* + *E* with $e_{ij}\sim N\left(0,\sigma^{2}\right)$. All the entries of design matrix *X* are generated as iid from *N*(0, 1). The true regression parameter Θ^*^ has a bi-cluster (checkerboard) structure. To simulate sparsity, we set the coefficients within many of the blocks in the checkerboard to 0. For the non-zero blocks, we follow the generative model recommended in Chi et al. (2014): the coefficients within each cluster are generated as θ_*ij*_ = μ_*rc*_ + ϵ_*ij*_ with $\epsilon_{ij}\sim N\left(0,\sigma_{\epsilon }^{2}\right)$ to make them close but not identical, where μ_*rc*_ is the mean of the cluster defined by the *rth* row partition and *cth* column partition. We set *n* = 50, *p* = 20, and *k* = 15. For the non-zero blocks, we set μ_*rc*_ ~ Uniform{−2, −1, 1, 2} and set σ_ϵ_ = 0.25. We set σ = 1.5. We use relatively small values for *p* and *k* since there will be a total of *pk* solution paths to visualize.

**Figure 3:**
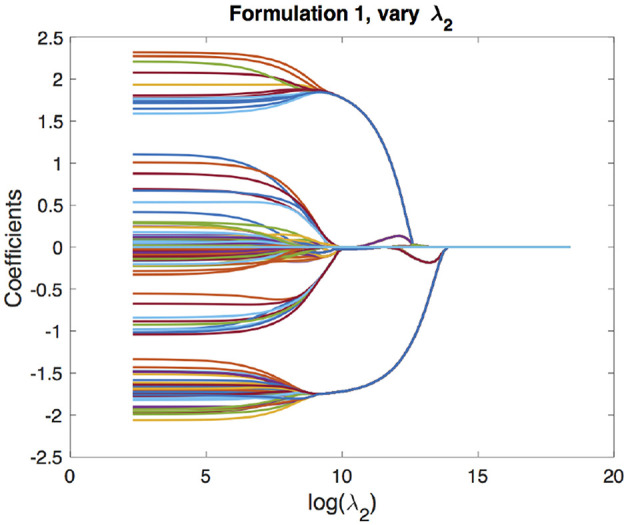
Solution paths for formulation 1, fixing λ_1_ and varying λ_2_. Each line indicates a distinct coefficient.

**Figure 4:**
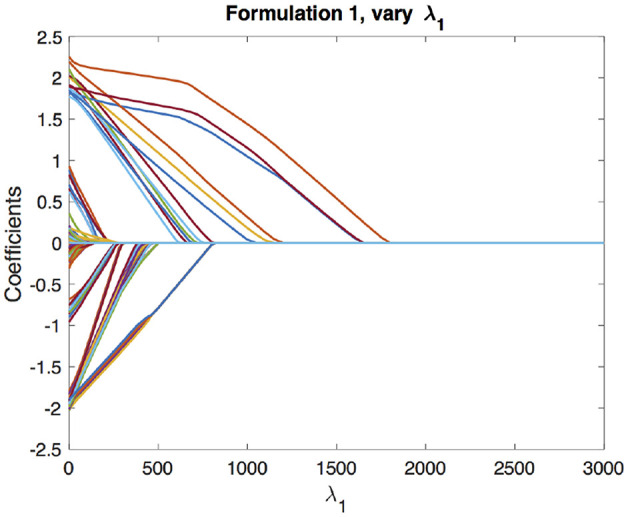
Solution paths for formulation 1, fixing λ_2_ and varying λ_1_. Each line indicates a distinct coefficient.

We begin with the solution paths for formulation 1. We first fix a reasonable λ_1_ and vary λ_2_ to get solution paths for all the coefficients. In our experiment, we chose λ_1_ based on cross-validation as described in section 4.1. These paths are shown in Figure 3. We can see that as λ_2_ increases, the coefficients begin to merge and eventually for large enough λ_2_ they are all equal. The solution paths are smooth in λ_2_. Similarly, we fix λ_2_ based on the cross-validation scheme described in section 4.2 and vary λ_1_ to get solution paths for all the coefficients. This is shown in Figure 4. It is well-known that the solution paths for LASSO are piecewise linear (Rosset and Zhu, 2007), when *L* is least squares loss. Here, we see that the solution paths are not piecewise linear, but rather a smoothed version of it. This smoothness is imparted by the convex clustering regularization, the third term in Equation (2).

**Figure 5:**
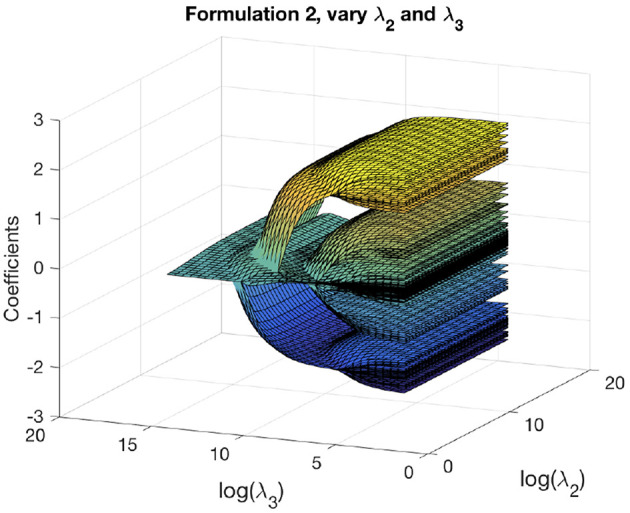
Solution paths for formulation 2, fixing λ_1_ and varying λ_2_, λ_3_.

**Figure 6:**
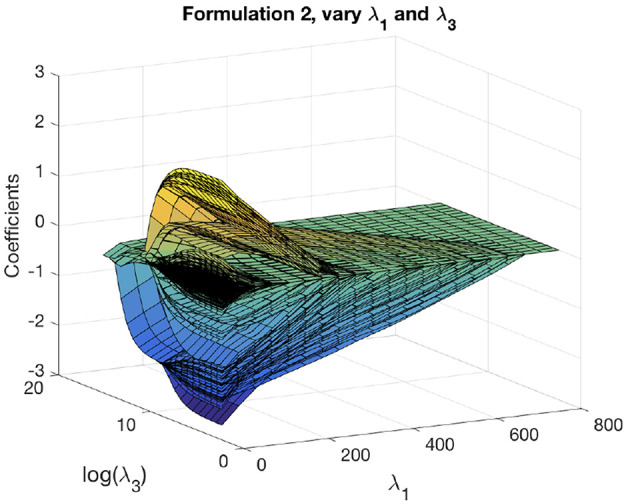
Solution paths for formulation 2, fixing λ_2_ and varying λ_1_, λ_3_.

We can obtain the solution path for formulation 2 as functions of two variables. We first fix a reasonable λ_1_ and vary λ_2_, λ_3_ to get solution paths for all the coefficients. These paths are shown in Figure 5. The solution paths are smooth in λ_2_ and λ_3_. Similarly, we fix a reasonable λ_2_ and vary λ_1_, λ_3_ to get solution paths for all the coefficients. These paths are shown in Figure 6. The solution paths are smooth in λ_1_ and λ_3_. The reasonable values are obtained using cross-validation.

### 4.4. Bi-clustering Thresholds

It is well-known that LASSO tends to select too many variables (Meinshausen and Yu, 2009). Hence ||Θ_·*i*_ − Θ_·*j*_||_2_ may not be exactly zero in most cases, and we may end up identifying too many clusters as well. In Chi et al. (2014) the authors defined the measure *v*_*ij*_ = ||Θ_·*i*_ − Θ_·*j*_||_2_ and placed the *ith* and *jth* columns in the same group if *v*_*ij*_ ≤ τ for some threshold τ, inspired by Meinshausen and Yu (2009). In our formulation 1, after selecting the best tuning parameters and estimating Θ, we place the *ith* and *jth* rows in the same group if ||Θ_*i*·_ − Θ_*j*·_||_2_ ≤ τ_*r*_. Similarly, if ||Θ_·*i*_ − Θ_·*j*_||_2_ ≤ τ_*c*_ we place the *ith* and *jth* columns in the same group. For formulation 2, we repeat the same approach using Γ instead of Θ.

To compute the thresholds τ_*r*_ and τ_*c*_, we first calculate [*v*_*col*_]_*ij*_ = ||Θ_·*i*_ − Θ_·*j*_||_2_ and stack this matrix to vector *v*_*col*_; similarly we calculate [*v*_*row*_]_*ij*_ = ||Θ_*i*·_ − Θ_*j*·_||_2_ and stack to vector *v*_*row*_. In the case of sparse linear regression, τ should be on the order of the noise (Meinshausen and Yu, 2009): $\tau \propto \sigma \sqrt{\log\left(p\right)/n}$, where σ is typically estimated using the standard deviation of residuals. In general we could set τ proportional to the standard deviation of *v*_*row*_ or *v*_*col*_.

However in our case, we have an additional regression loss term for estimating the parameters and hence there are two sources of randomness, the regression residual and the error in *v*. Taking these into account, we set $\tau_{c}=\frac{1}{2}[\sigma \sqrt{\log\left(p\right)/n}+std\left(v_{col}\right)]$ and $\tau_{r}=\frac{1}{2}[\sigma \sqrt{\log\left(p\right)/n}+std\left(v_{row}\right)]$. We set the multiplier to $\frac{1}{2}$, following the usual conservative heuristics.

### 4.5. Specializing to Column- or Row-Only Clustering (a.k.a. *Uni-clustering*)

Although formulations 1 and 2 have been developed for row-column bi-clustering, they can be easily specialized to clustering columns or rows alone, by respectively using only Ω_*W*_(Θ) or $\Omega_{\tilde{W}}\left(\Theta^{T}\right)$ in Equation (2), or using only Ω_*W*_(Γ) or $\Omega_{\tilde{W}}\left(\Gamma^{T}\right)$ in Equation (3).

## 5. Synthetic Data Experiments

We demonstrate our approach using experiments with synthetic data on the problem of multi-task learning. *As emphasized before, our main focus is on bi-clustering result instead of parameter estimation*. We begin by describing the performance measures used to evaluate the clustering and estimation performance.

### 5.1. Performance Measures

Assessing the clustering quality can be hard. In this paper, we use the following three measures to evaluate the quality of clustering: the adjusted Rand index (Hubert and Arabie, 1985) (ARI), the F-1 score (F-1), and the Jaccard index (JI).

Assume B is the true clustering, define *TP* to be the number of pairs of elements in *S* that are in the same subset in A and in the same subset in B. This is the true positive and similarly we can define *TN*, *FN*, *FP* as true negative, false negative, and false positive, respectively. Define $precision=\frac{TP}{TP+FP}$ and $recall=\frac{TP}{TP+FN}$, the F-1 score is defined as:

(7) $$\begin{array}{r} \text{F-1}=\frac{2\cdot precision\cdot recall}{precision+recall} \end{array}$$

Using the same notation as F-1 score, the Jaccard Index is defined as:

(8) $$\begin{array}{r} \text{JI}=\frac{TP}{TP+FP+FN} \end{array}$$

For all these three measures, a value of 1 implies the best possible performance, and a value of 0 means that we are doing poorly. In order to compute ARI, F-1, and JI, we choose the value of the multiplier λ_2_ in formulation 1, and {λ_2_, λ_3_} in formulation 2 using the approach described in section 4.2, and obtain the estimated clusterings.

The estimation accuracy is measured by calculating the RMSE on an independent test set, and also the parameter recovery accuracy, $\left\|{\hat{\Theta }}_{est}-\Theta^{*}\|/\|\Theta^{*}\right\|$ where ${\hat{\Theta }}_{est}$ and Θ^*^ are the estimated and true coefficient matrices.

### 5.2. Simulation Setup and Results

We focus on multi-task regression: *Y* = *X*Θ* + *E* with $e_{ij}\sim N\left(0,\sigma^{2}\right)$. All the entries of design matrix *X* are generated as iid from *N*(0, 1). The true regression parameter Θ^*^ has a bi-cluster (checkerboard) structure. To simulate sparsity, we set the coefficients within many of the blocks in the checkerboard to 0. For the non-zero blocks, we follow the generative model recommended in Chi et al. (2014): the coefficients within each cluster are generated as θ_*ij*_ = μ_*rc*_ + ϵ_*ij*_ with $\epsilon_{ij}\sim N\left(0,\sigma_{\epsilon }^{2}\right)$ to make them close but not identical, where μ_*rc*_ is the mean of the cluster defined by the *r*^*th*^ row partition and *c*^*th*^ column partition. We set *n* = 200, *p* = 500, and *k* = 250 in our experiment. For the non-zero blocks, we set μ_*rc*_ ~ Uniform{−2, −1, 1, 2} and set σ_ϵ_ = 0.25. We try the low-noise setting (σ = 1.5), where it is relatively easy to estimate the clusters, and the high-noise setting (σ = 3), where it is harder to obtain them.

We compare our formulations 1 and 2 with a 2-step *estimate-then-cluster* approach: (a) Estimate $\hat{\Theta }$ first using LASSO, and (b) perform convex bi-clustering on $\hat{\Theta }$. $\hat{\Theta }$ is estimated by solving (6) while selecting the best λ_1_ as discussed in section 4.1, and the convex bi-clustering step is implemented using COBRA algorithm in Chi et al. (2014). Our *baseline* clustering performance is the best of: (a) letting each coefficient be its own group, and (b) imposing a single group for all coefficients.

The average clustering quality results on 50 replicates are shown in Table 1 for low and high noise settings. Most performance measures are reported in the format *mean*±*std*.*dev*. In both tables, the first, second, and third blocks correspond to performances of row, column and row-column bi-clusterings, respectively. We optimize only for bi-clusterings, but the row and the column clusterings are obtained as by-products.

**Table 1:** Performance of low and high noise settings.

**Noise**	**Metric**	**Baseline**	**2-step**	**F1**	**F2**
Low	ARI	0	0.679 ± 0.157	0.869 ± 0.069	0.900 ± 0.046
Low	F-1	0.446	0.757 ± 0.128	0.907 ± 0.052	0.931 ± 0.022
Low	JI	0.287	0.625 ± 0.161	0.834 ± 0.081	0.871 ± 0.042
Low	ARI	0	0.877 ± 0.043	0.914 ± 0.020	0.915 ± 0.013
Low	F-1	0.446	0.908 ± 0.037	0.933 ± 0.023	0.934 ± 0.012
Low	JI	0.287	0.847 ± 0.048	0.876 ± 0.031	0.887 ± 0.025
Low	ARI	0	0.708 ± 0.118	0.841 ± 0.059	0.863 ± 0.035
Low	F-1	0.172	0.734 ± 0.110	0.857 ± 0.052	0.877 ± 0.026
Low	JI	0.094	0.591 ± 0.134	0.753 ± 0.077	0.781 ± 0.035
High	ARI	0	0.577 ± 0.163	0.803 ± 0.104	0.804 ± 0.096
High	F-1	0.446	0.674 ± 0.138	0.874 ± 0.093	0.874 ± 0.075
High	JI	0.287	0.525 ± 0.159	0.793 ± 0.097	0.792 ± 0.098
High	ARI	0	0.734 ± 0.132	0.905 ± 0.077	0.905 ± 0.046
High	F-1	0.446	0.799 ± 0.107	0.924 ± 0.054	0.933 ± 0.039
High	JI	0.287	0.689 ± 0.120	0.872 ± 0.078	0.867 ± 0.065
High	ARI	0	0.555 ± 0.187	0.801 ± 0.125	0.812 ± 0.105
High	F-1	0.172	0.586 ± 0.152	0.824 ± 0.104	0.821 ± 0.086
High	JI	0.094	0.437 ± 0.179	0.714 ± 0.118	0.713 ± 0.104

*For each noise setting, the first block is for row clustering; the second block is for column clustering; and the third block is for row-column biclustering*.

From Table 1 we see that both our formulation 1 and 2 give better results on row clustering, column clusterings, and row-column bi-clustering compared to the 2-step procedure. Moreover, the clustering results given by our formulations are more stable, with lesser spread in performance.

It is also instructive to note that the performance obtained for columns is substantially higher compared to those obtained with rows. This could be because of two reasons: (a) the columns of Θ have a one-to-one correspondence to the columns of the task responses *Y*, and hence any relationship between the tasks is easily inherited, (b) the rows of Θ can be noisier than the columns, since each row contributes to all the tasks.

The performance boost obtained with high noise is much higher compared to that with low noise. This makes sense because when noise level is low, the estimation step in the 2-step approach is more accurate and the error propagated into the clustering step is relatively small. However, at high noise levels, the estimation can be inaccurate. This estimation error propagates into the clustering step and makes the clustering result of 2-step approach unreliable. Since our formulations jointly perform estimation and clustering, they obtain more reliable and stable results.

The RMSEs evaluated on the test set and the parameter recovery accuracy are provided in Table 2. The oracle RMSE (with Θ known) is 1.5 for the low noise setting and 3.0 for the high noise setting in Table 2, and we can see that the proposed methods provide improvements over the others. We also observe improvements in the parameter recovery accuracy. Although the improvement is marginal, it demonstrates that we are not losing estimation accuracy because of the biclustering structure we considered.

**Table 2:** RMSE and parameter recovery accuracy of the estimation schemes for low noise (σ = 1.5) and high noise (σ = 3) settings.

**Noise**	**Accuracy metric**	**Lasso**	**2-step**	**Form1**	**Form2**
Low	RMSE	1.627 ± 0.02	1.622 ± 0.02	1.613 ± 0.02	1.612 ± 0.02
Low	Rec. acc.	0.234 ± 0.03	0.231 ± 0.03	0.223 ± 0.03	0.222 ± 0.03
High	RMSE	3.34 ± 0.02	3.30 ± 0.02	3.23 ± 0.02	3.16 ± 0.02
High	Rec. acc.	0.364 ± 0.06	0.362 ± 0.06	0.327 ± 0.05	0.325 ± 0.06

## 6. Real Data Experiments

We demonstrate the proposed approaches using real datasets obtained from experiments with Sorghum crops (Tuinstra, 2016). We consider two specific problems from this pipeline: (a) predictive modeling of plant traits using features from remote sensed data (section 6.1), (b) GWAS using the reference traits (section 6.2).

### 6.1. Phenotypic Trait Prediction From Remote Sensed Data

The experimental data was obtained from 18 Sorghum varieties planted in 6 replicate plot locations, and we considered the trait of plant height. The 18 variety names are given in the Supplementary Material.

From the RGB and hyperspectral images of each plot, we extract features of length 206. Hence *n* = 6, *p* = 206, and the number of tasks *k* = 18, for each trait considered. The presence of multiple varieties with replicates much smaller in number than predictors poses a major challenge: building separate models for each variety is unrealistic, while a single model does not fit all. This is where our proposed simultaneous estimation and clustering approach provides the flexibility to share information among tasks that leads to learning at the requisite level of robustness. Note that here we use the column-only clustering variant of formulation 1.

**Figure 7:**
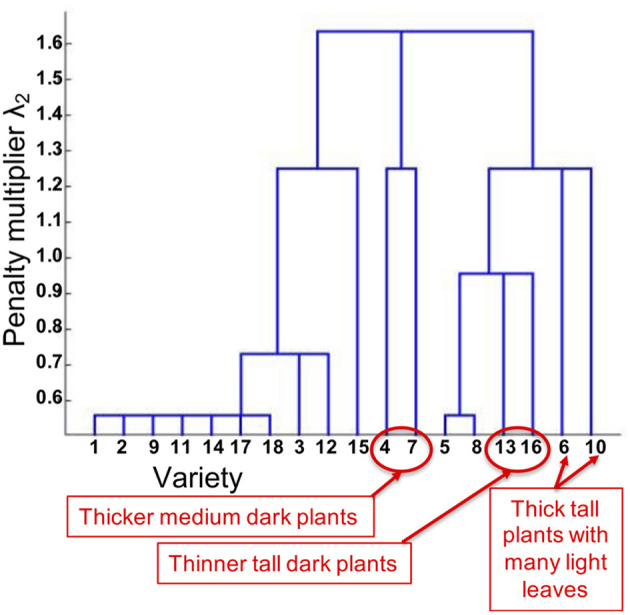
Tree structure of tasks (varieties) inferred using our approach for plant height.

The dendrogram for task clusters obtained by sweeping the penalty multiplier λ_2_ is given in Figure 7. This provides some interesting insights from a plant science perspective. As highlighted in Figure 7, the predictive models (columns of Θ) for thicker medium dark plants are grouped together. Similar grouping is seen for thinner tall dark plants, and thick tall plants with many light leaves.

To compute RMSE, we perform 6-folds CV where each fold consists of at least one example from each variety. As we only have *n* = 6 samples per variety (i.e., per task), it is unrealistic to learn separate models for each variety. For each CV split, we first learn a grouping using one of the compared methods, treat all the samples within a group as i.i.d, and estimate their regression coefficients using Lasso. The methods compared with our approach include: (a) *single model*, which learns a single predictive model using Lasso, treating all the varieties as i.i.d., (b) *No group multitask learning*, which learns a traditional multitask model using Group Lasso where each variety forms a separate group, and (c) Kang et al. (2011), which uses a mixed integer program to learn shared feature representations among tasks, while simultaneously determining “with whom” each task should share. Results reported in Table 3, indicate the superior quality of our groupings in terms of improved predictive accuracy.

**Table 3:** RMSE for plant height prediction.

**Method**	**RMSE**
Single model	44.39 ± 6.55
No group multitask learning	36.94 ± 6.10
Kang et al.	37.55 ± 7.60
Proposed	**33.31** ± **5.10**

*The bold value indicates root mean square error*.

### 6.2. Multi-Response GWAS

We apply our approach in a multi-response Genome-Wide Association Study (GWAS). While traditional GWAS focuses on associations to single phenotypes, we would like to automatically learn the grouping structure between the phenotypes as well as the features (columns and rows of Θ) using our proposed method. We use the proposed formulations 1 and 2 (bi-clustering variant) in this experiment.

The design matrix *X* consisted of SNPs of Sorghum varieties. We consider *n* = 911 varieties and over 80,000 SNPs. We remove duplicate SNPs and also SNPs that do not have significantly high correlation to at least one response variable. Finally, we end up considering *p* = 2,937 SNPs. The output data *Y* contains the following 6 response variables (columns) for all the *n* varieties collected by hand measurements:

1. *Height to panicle* (h1): The height of the plant up to the panicle of the Sorghum plant.
2. *Height to top collar* (h2): The height of the plant up to the top most leaf collar.
3. *Diameter top collar* (d1): The diameter of the stem at the top most leaf collar.
4. *Diameter at 5 cm from base* (d2): The diameter of the stem at 5 cm from the base of the plant.
5. *Leaf collar count* (l1): The number of leaf collars in the plant.
6. *Green leaf count* (l2): The total number of green leaves. This will be <l1 since some leaves may have senesced and will not be green anymore.

For each variety, each trait can be an average of measurements from up to four plants.

**Figure 8:**
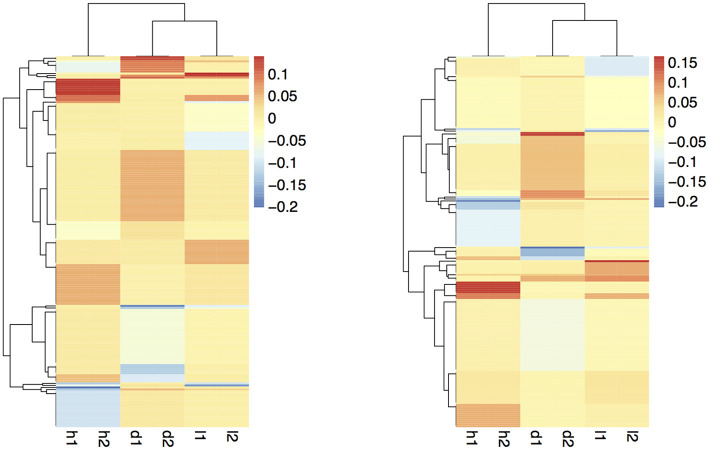
Smoothed coefficient matrix obtained from formulations 1 (left) and 2 (right), revealing the bi-clustering structure.

The coefficient matrix given by our formulations are visualized in Figure 8. To make the figure easier to interpret, we exclude the rows with all zero coefficients and take the average over the coefficients within each bi-cluster. The light yellow regions are coefficients close to zero; red and blue areas are positive and negative coefficients, respectively. The rows and columns are reordered to best show the checkerboard patterns. We wish to emphasize again that these checkerboard patterns in the coefficient matrices are automatically discovered using our proposed procedures, and are not readily evident, or trivially discoverable from the data.

**Figure 9:**
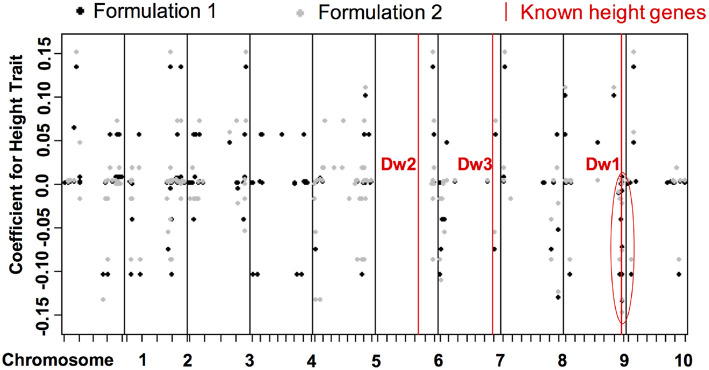
Distribution of coefficients for height traits for all SNPs. The x-axis shows the positions of genetic variants on the chromosomes. The y-axis are the values of the coefficients for the discovered associations with height trait.The red lines are loci of known height genes, namely genes that are known to be associated to height, and the black and gray dots correspond to coefficients of formulations 1 and 2, respectively. Some correspond to known locations, some correspond to new locations of associated SNPS.

The two formulations reveal similar bi-clustering patterns up to reordering. For column clusters, the plant height tasks (h1 and h2), the stem diameter tasks (d1 and d2), and the leaf tasks (l1 and l2) group together. Also, the stem diameter and leaf tasks are more related to each other compared to the height tasks. The bi-clustering patterns reveal the groups of SNPs that influence similar phenotypic traits. Coefficients for height features in the GWAS (Figure 9) study show SNPs with strong effects coinciding with locations of Dwarf 3 (Multani et al., 2003) and especially Dwarf 1 (Hilley et al., 2016) genes known to control plant height that are segregating and significant in the population. The lack of any effect at the Dwarf 2 (Hilley et al., 2017) locus supports previous work indicating that this gene is not a strong contributing factor in this population. This demonstrates that we are able to discover existing factors. We also identify potentially new SNPs for further investigation and biological validation, since many coefficients align with loci outside of the previously identified known height genes.

To evaluate predictive accuracy, we split our data set into three parts: 70% training, 15% validation, and 15% test. We estimate the coefficient matrices by optimizing our formulations on the training set, select the tuning parameters based on the validation set (sections 4.2, 4.4), and then calculate the RMSE on the test set. Table 4 shows the RMSE on test set.

**Table 4:** Comparison of test RMSE on the multi-response GWAS dataset.

	**Lasso**	**2-step**	**Form1**	**Form2**
RMSE	2.181	2.206	2.105	2.119

We also estimate the RMSE of the proposed formulations and compare it with the RMSE provided by a simple Lasso model and 2-step procedure. This is shown in Table 4. We see that the RMSE of our formulations are slightly less than that of the Lasso and 2-step procedure. Hence, for similar estimation performance, we are able to discover additional interesting structure in the input-output relationship using our proposed methods.

## 7. Concluding Remarks

In this paper we introduced and studied formulations for joint estimation and clustering (row or column or both) of the parameter matrix in multi-response models. By design, our formulations imply that coefficients belonging to the same (bi-)cluster are close to one another. By incorporating different notions of closeness between the coefficients, we can tremendously increase the scope of applications in which similar formulations can be used. Some future applications could include sparse subspace clustering and community detection.

Recently there has been a lot of research on non-convex optimization formulations, both from theoretical and empirical perspectives. It would be of interest to see the performance of our formulations on non-convex loss functions. Another extension would be to construct confidence intervals and perform hypothesis testing for the coefficients in each cluster.

## Data Availability

The raw data supporting the conclusions of this manuscript will be made available by the authors, without undue reservation, to any qualified researcher.

## Author Contributions

MY discussed the idea, wrote the codes, ran the experiments, and wrote the second part of the paper. KN discussed the idea and wrote the first part of the paper. AT provided the real data and interpreted the real data experiments. AL proposed the original research idea, and wrote the first part of the paper.

### Conflict of Interest Statement

KN and AL were employed by IBM. The remaining authors declare that the research was conducted in the absence of any commercial or financial relationships that could be construed as a potential conflict of interest.
