# Supplementary material for: Simultaneous Parameter Learning and Bi-clustering for Multi-Response Models
Source: Front Big Data. 2019 Aug 14;2:27. doi: 10.3389/fdata.2019.00027 (PMC7931892; doi:10.3389/fdata.2019.00027)
Supplement: Supplementary file 1 [file Data_Sheet_1.pdf]

SUPPLEMENTARY MATERIAL

This material provides additional details to support the main paper.

COnvex BiclusteRing Algorithm (COBRA) Chi et al. (2014)

We provide the details of the COnvex BiclusteRing Algorithm (COBRA) introduced in Chi et al. (2014) for completeness.

Algorithm 3: Convex biclustering (COBRA) Chi et al. (2014)

Result: Estimated Γ
Initialize $\Gamma_0 = \Theta$, $P_0 = 0$, $Q_0 = 0$, iteration $m = 0$
while *not converged* **do**
  $Y_m = \text{prox}_{\frac{\lambda_3}{\lambda_2}\Omega_{\widetilde{W}}}(\Gamma_m^T + P_m^T)$ (row clustering)
  $P_{m+1} = \Gamma_m + P_m - Y_m^T$
  $\Gamma_{m+1} = \text{prox}_{\frac{\lambda_3}{\lambda_2}\Omega_W}(Y_m^T + Q_m^T)$ (col. clustering)
  $Q_{m+1} = Y_m + Q_m - \Gamma_{m+1}^T$
  $m = m + 1$
end

Proof of Proposition 1

PROOF. We need to check that the conditions in Theorem 3.4 in Combettes and Pesquet (2008) are satisfied in our case:

$$\text{(i)} \lim_{\|\Theta\|\to+\infty} f_1(\Theta) + f_2(\Theta) + f_3(\Theta) = +\infty$$

$$\text{(ii)}(0,\dots,0) \in \text{sri}\{(\Theta-\Theta_1,\Theta-\Theta_2,\Theta-\Theta_3)|\Theta\in\mathbb{R}^{pk},$$
$$\Theta_1 \in \text{dom} f_1, \Theta_2 \in \text{dom} f_2, \Theta_3 \in \text{dom} f_3\}$$

Let $\mathcal{H}$ be the domain of Θ which can be set as $\mathbb{R}^{pk}$. Let C be a nonempty convex subset of $\mathcal{H}$, the strong relative interior of C is

$$\text{sri}(C) = \left\{\Theta \in C|\text{cone}(C-\Theta) = \overline{\text{span}}(C-\Theta)\right\}$$

where $\text{cone}(C) = \bigcup_{\lambda>0}\{\lambda\Theta|\Theta\in C\}$, and $\overline{\text{span}}(C)$ is the closure of span C.

Now we check the conditions. For (i), $\|\Theta\|$ goes to infinity means some $\|\Theta_s\|$ goes to infinity, and then we know f_2 goes to infinity. Therefore (i) holds.

For (ii), we do not have any restriction on Θ, so the right hand side is just $\text{sri}(\mathbb{R}^{pk})$, hence (ii) holds.

Therefore, the proposition follows according to Theorem 3.4 of Combettes and Pesquet (2008).

Proof of Proposition 2

PROOF. The optimization step for Γ is solved by COBRA and it converges to global minimizer according to Proposition 4.1 in Chi et al. (2014). Define $f(\Theta,\Gamma) = \lambda_2\sum_{i=1}^k \|\Theta_i - \Gamma_i\|_2^2$ with $\nabla f_\Theta(\Theta,\Gamma) =$

Table 5. RMSE for different comparison methods

Method	RMSE	std	time	RMSE(close)
Single task Lasso	1.665	0.018	0.108	1.894
No group multitask learning	1.799	0.016	1.079	1.993
Kang et al	2.015	0.016	56.07	2.116
Tree guided group Lasso	1.655	0.018	0.352	1.875
F_1	**1.635**	0.015	2.892	**1.757**
F_2	**1.647**	0.017	2.350	**1.736**

$2\lambda_2(\Theta - \Gamma)$ and $\nabla f_\Gamma(\Theta, \Gamma) = 2\lambda_2(\Gamma - \Theta)$, it is clear that $\nabla f_\Theta(\Theta, \Gamma)$ and $\nabla f_\Gamma(\Theta, \Gamma)$ are both Lipschitz-continuous in Θ and Γ, respectively. Since the optimization step for Θ is also assumed to find global minimizer, Theorem 3.9 in Beck (2015) guarantees that our algorithm in Section 3.2 converges to the global minimizer.

Simulation experiments on uni-clustering

We evaluate the performance of our proposed method on synthetic datasets and compare with the following existing methods:

- **Single task**: a baseline approach, where the tasks are learned separately via Lasso.
- **No group MTL** Obozinski et al. (2006): the traditional multitask approach using group lasso penalty, where all tasks are learned jointly and the same features are selected across tasks.
- **Kang et al** Kang et al. (2011): Mixed integer program learning a shared feature representations among tasks, while simultaneously determining “with whom” each task should share. We used the code provided by the authors of Kang et al. (2011) and used the true number of tasks.
- **Tree-guided group Lasso** Kim and Xing (2010): Employs a structured penalty function induced from a predefined tree structure among responses, that encourages multiple correlated responses to share a similar set of covariates. We used the code provided by the authors of Kim and Xing (2010) where the tree structure is obtained by running a hierarchical agglomerative clustering on the responses.

Since all of these methods focus on estimation accuracy, we compare the root mean square error (RMSE) only. The parameter settings are the same as before. Columns 2-4 in Table 5 shows the comparison results on RMSE, standard deviation of RMSE, and running time. From Table 5 we see that we obtain slightly improved RMSE (recall that the grouping quality is significantly improved) and our algorithm remains efficient in high dimensions.

To show further the gain of our proposed formulation, we consider a slightly different setting where we set $\sigma_\epsilon = 0.05$ such that the parameters within groups are more close to each other. We also increase the number of nonzero components in the true coefficient matrix so that the estimation problem is much more difficult (i.e. a larger RMSE). The last column of Table 5 shows the RMSE under this setting, where we see that our proposed formulations improve the estimation accuracy.

Real data setting

The varieties and their names used in the trait prediction experiments (Section 6.1) are given in Table 6.

Variety number	Variety name
1	RS 392x105 BMR FS
2	RS 400x38 BMR SG
3	RS 341x10 FG white
4	RS 374x66 FS
5	RS 327x36 BMR FS
6	RS 400x82 BMR SG
7	RS 366x58 FG white
8	SP NK5418 GS
9	SP NK8416 GS
10	SP SS405 FS
11	SP Trudan Headless FS PS
12	SP Trudan 8 FS
13	SP HIKANE II FS
14	SP NK300 FS
15	SP Sordan Headless FS PS
16	SP Sordan 79 FS
17	PH 849F FS
18	PH 877F FS

Table 6. Numbers and names of Sorghum varieties used in experiments.
